# Occurrence of *Aspergillus* spp. in Parrot Feeds on the Polish Market: The Potential Health Threat of Aspergillosis and Mycotoxicosis for Exotic Pet Birds, a Pilot Study

**DOI:** 10.3390/vetsci12060597

**Published:** 2025-06-18

**Authors:** Aleksandra Kornelia Maj, Piotr Górecki, Olga Szaluś-Jordanow, Dawid Jańczak

**Affiliations:** 1 Animallab Veterinary Laboratory, Środkowa 2/4, 03-430 Warsaw, Poland; aleksandra.kornelia.maj@gmail.com (A.K.M.);; 2 Department of Small Animal Diseases with Clinic, Institute of Veterinary Medicine, Warsaw University of Life Sciences-SGGW, Nowoursynowska 159c, 02-776 Warsaw, Poland; 3 Department of Infectious and Invasive Diseases and Veterinary Administration, Institute of Veterinary Medicine, Faculty of Biological and Veterinary Sciences, Nicolaus Copernicus University, Gagarina 7, 87-100 Toruń, Poland

**Keywords:** parrots, *Aspergillus* spp., parrot feed, contamination, mycotoxins, aspergillosis, Poland

## Abstract

A lack of awareness among exotic bird owners regarding the quality of feed may contribute to adverse health outcomes, including toxicosis, systemic mycoses, and potentially neoplastic processes. Fungi of the *Aspergillus* genus are the most pathogenic to avian species, particularly due to their involvement in respiratory diseases such as aspergillosis, which affects the air sacs. This study aims to assess the presence of *Aspergillus* spp. in commercially available parrot feed (grain mixtures) available on the Polish pet market, considering different price categories. A total of 22 dry parrot food samples were analyzed using the PN-ISO 21527-2:2009 protocol. *Aspergillus* spp. colonies were isolated from 16 out of 22 samples (72.7%), indicating a high incidence of contamination. Although these results are preliminary, they highlight a microbiological risk associated with grain-based parrot feeds and underscore the need for stricter quality control and greater awareness among pet owners and manufacturers.

## Simple Summary

Many people feed their pet parrots with commercially available grain mixtures, assuming they are safe and healthy. However, our study found that these feeds can be contaminated with mold, including fungi that may cause serious respiratory illness in birds. We examined 22 popular parrot feeds sold in Poland and found mold in nearly three out of four samples. The most common fungi belonged to the group known as *Aspergillus*, which can lead to dangerous infections in birds. One of the species we found, *Aspergillus fumigatus*, is especially harmful because its tiny spores can access deep areas of a bird’s lungs. Surprisingly, we also found mold in some more expensive feeds, showing that a higher price does not always mean better quality. Currently, there are no clear safety rules or limits for mold in parrot feed. Our findings suggest that there is an urgent need for better control of feed production and greater awareness among parrot owners, breeders, and veterinarians. Feeding pet birds clean, high-quality food is essential for protecting their health and well-being. We hope this study encourages further research and leads to the development of safety standards for bird feed.

## 1. Introduction

Birds display greater sensitivity to zoo-hygienic conditions than mammals, necessitating distinct preventive and clinical approaches in avian medicine. This susceptibility stems from unique anatomical and physiological traits. Additionally, their inborn tendency to mask clinical signs of illness, an evolutionary adaptation to avoid predation or exclusion from the flock, often delays diagnosis and complicates timely therapeutic intervention [1]. In addition to this behavioral adaptation, the avian respiratory system is characterized by a highly efficient yet delicate architecture. Birds possess a complex system of air sacs that extend throughout the body and connect to the lungs, facilitating unidirectional airflow and exceptionally efficient gas exchange. However, this anatomical specialization also renders birds particularly vulnerable to airborne contaminants, including dust particles, fungal spores, and gaseous toxins [2]. Daily inhalation of fungal spores or exposure to secondary metabolites such as mycotoxins, sometimes even from fungal species generally regarded as saprophytic, can result in the development of overt clinical disease. These exposures may lead to a progressive decline in respiratory function, general debilitation, and in severe cases, mortality [3,4].

Among the fungal diseases affecting avian species, aspergillosis is one of the most prevalent and clinically significant. The condition is most frequently associated with *Aspergillus* fumigatus, a ubiquitous environmental mold that thrives in warm, moist, and organic-rich environments such as bedding, feed, and nesting material [5]. However, a variety of other *Aspergillus* species, including *A. niger*, *A. flavus*, *A. glaucus*, and *A. parasiticus*, have also been identified in clinical cases. In some instances, mixed-species infections further complicate the clinical picture, particularly among poultry such as chickens and ostriches, companion birds including parrots, and numerous species of wild birds [4,6,7,8,9,10,11]. The predominance of *A. fumigatus* in avian aspergillosis is often attributed to the small size of its conidia (spores), which enhances their aerodynamic properties and allows for deep penetration into the distal regions of the avian respiratory tract. This facilitates colonization of the air sacs and lung parenchyma, initiating a pathogenic cascade that can be difficult to halt once established [12,13].

A notable event highlighting the impact of *Aspergillus* spp. on avian health and feed safety occurred in 1960 during the “Turkey X Disease” outbreak in the UK. The mortality was traced to *A. flavus* contaminated Brazilian groundnut meal, which contained hepatotoxic and immunosuppressive aflatoxins [14]. This incident marked a turning point in mycotoxicology and spurred the development of global regulatory frameworks for mycotoxin control in animal feed. Importantly, fungal growth in feed does not always correlate with mycotoxin production, as toxin synthesis depends on environmental factors such as temperature, humidity, oxygen, substrate, and fungal strain variability [15]. Consequently, feed may appear visually sound while harboring dangerous levels of mycotoxins, or conversely, exhibit mold growth without measurable toxin levels. Among the wide array of mycotoxin-producing fungi, several species have emerged as particularly significant due to the potency and prevalence of the toxic metabolites they produce. The most important species within the genus *Aspergillus* responsible for the biosynthesis of aflatoxins (AF) include *A. flavus*, *A. parasiticus*, *A. nomius*, and *A. pseudotamarii* [16,17,18,19,20]. These aflatoxins are among the most studied and highly toxic mycotoxins, with aflatoxin B_1_ being classified as a Group 1 carcinogen by the International Agency for Research on Cancer (IARC) [21]. In addition, *A. ochraceus* and *A. sulphureus*, alongside *Penicillium verrucosum*, are notable for their ability to synthesize ochratoxins (OT), which are nephrotoxic and potentially carcinogenic compounds [16,17,18,19,20]. Meanwhile, *Fusarium* species commonly encountered in both pre- and post-harvest plant material are primarily responsible for the production of fumonisins and zearalenone (ZEA), which exert hepatotoxic, nephrotoxic, and estrogenic effects [16,17,18,19,20]. These fungi have a high capacity to colonize a wide range of agricultural crops both during plant development and after harvest. Once established on a substrate, they may persist and continue to produce mycotoxins throughout the processing and storage phases. Crops such as cereals (e.g., maize, wheat, barley), legumes, nuts, vegetables, and fruits are particularly susceptible to fungal contamination, especially under inadequate storage conditions [22]. The contamination is not restricted to the raw material; animal feed derived from such ingredients can also harbor active mycotoxins or their degradation products. A particularly insidious threat in the context of food and feed safety is posed by so-called “masked” mycotoxins. These compounds are formed when mycotoxins are conjugated with plant-derived molecules or modified during metabolic processes, rendering them less detectable by standard analytical methods. Despite undergoing physical, thermal, or chemical treatments such as drying, pelleting, or extrusion, these masked mycotoxins often remain intact and biologically active. Their detection and quantification thus represent a major challenge in food safety monitoring and toxicological risk assessment [23]. The toxic agent responsible for the 1960 “Turkey X Disease” was identified as aflatoxin, a discovery foundational to the understanding of aflatoxicosis [24]. Aflatoxins exhibit hepatotoxic and carcinogenic effects in multiple avian species and mammals, with characteristic hepatic lesions such as fatty degeneration, bile duct proliferation, and hepatic carcinoma [24,25]. Beyond animal health, mycotoxins and their metabolites can accumulate in edible products, posing risks to human consumers [26]. Consequently, comprehensive mycotoxin monitoring throughout the feed and food chain is essential for both animal and public health protection [27].

Although clinical aspergillosis is commonly presumed to be prevalent among avian species, it is likely underdiagnosed, largely due to the inherent challenges of clinical identification. In ornamental birds, diagnosis often relies on owner-provided history particularly dietary information combined with clinical signs, radiographic findings, and quantification of galactomannan antigen, a polysaccharide component of the *Aspergillus* cell wall, in blood samples [28]. Traditional microbiological swabs from the upper respiratory tract offer limited utility due to the environmental ubiquity of *Aspergillus* species and the high risk of false-positive results [29]. More reliable diagnostic specimens include those obtained via endoscopic inspection of the air sacs or, alternatively, through exploratory laparotomy for internal organ biopsies [30]. Serological testing for anti-*Aspergillus* antibodies may be employed; however, its diagnostic value is similarly compromised by the fungus’s widespread environmental presence [31,32]. While the respiratory system is the primary site affected in avian aspergillosis, systemic dissemination to other organs is also possible [8]. The disease may manifest as a chronic, slowly progressive form or an acute, rapidly fatal condition, particularly in the context of mycotoxicosis. Continuous inhalation of high spore loads and compromised immune status are key predisposing factors. Susceptibility to fungal infection and the toxic effects of aflatoxins vary depending on species, age, immune competence, and composition of the diet [33,34,35]. Notably, birds fed protein-deficient diets such as typical seed mixtures formulated for parrots are significantly more vulnerable to aflatoxin-induced damage compared to animals receiving nutritionally balanced rations [36]. Additional nutritional inadequacies, including hypovitaminosis A and epithelial barrier dysfunction, further heighten disease susceptibility [37,38]. The present study aims to investigate the presence of *Aspergillus* fungi in loose feed formulations (grain mixtures) intended for parrots and commercially available on the Polish pet market, as part of a pilot investigation conducted in Poland.

## 2. Materials and Methods

In the present study, a total of 22 commercially available parrot feed products were subjected to analysis. To minimize potential bias associated with individual brands or manufacturers and to ensure neutrality in interpretation, the feeds were stratified into four categories according to their retail price per kilogram. The price ranges were defined as follows: less than 4.5 EUR/kg, between 4.5 and 6.9 EUR/kg, between 7.0 and 20.0 EUR/kg, and above 20.0 EUR/kg.

Only feeds that were commercially pre-packaged and hermetically sealed by the manufacturer were included in the study. To ensure a random and unbiased selection, products were chosen without preference for brand, manufacturer, or composition. Each sample remained unopened and stored in its original airtight packaging until the time of analysis, thereby minimizing environmental exposure and preserving the integrity of the product.

Immediately prior to testing, the packaging of each feed sample was carefully opened, and an initial qualitative assessment of its physical properties was conducted. This evaluation included a macroscopic examination of texture, homogeneity, and the presence of any visible contaminants, as well as an organoleptic assessment of odor. Importantly, none of the tested feeds exhibited any perceptible moldy, musty, or otherwise unpleasant smell that could be indicative of fungal contamination or spoilage.

Of the 22 feed samples analyzed, 20 were sourced from physical retail pet stores located throughout various regions of Poland. These included both chain and independent pet supply outlets. The remaining two samples, corresponding to the most expensive products within the study group were obtained via an established online pet food retailer. This distinction in purchase method was solely due to the limited physical availability of high-end feeds within brick-and-mortar stores and was not reflective of any experimental preference.

For statistical analysis, the chi-square test was employed to assess the relationship between mold growth and feed moisture content, as well as the potential association between feed price and the presence of fungal contamination. There is currently no standardized research methods specifically designed for detecting *Aspergillus* spp. in animal feed. Therefore, a general method for the enumeration of yeasts and molds was employed, in accordance with PN-ISO 21527-2:2009 [39]. The analysis was conducted using the plate count method on Sabouraud Dextrose Agar supplemented with gentamicin and chloramphenicol (GRASO Biotech, Starogard Gdański, Poland). Prior to inoculation, any condensation was removed from the surface of the agar plates by drying them in an incubator at 40 °C for 30 min. During this process, plates were inverted to prevent contamination from airborne fungal spores. For each sample, 10 g of parrot feed was mixed with 90 mL of sterile physiological saline solution. The mixture was then homogenized mechanically. From each dilution, 1 mL was taken and serially diluted to concentrations of 10^−2^ and 10^−3^. From each dilution, 100 µL was plated onto the surface of Sabouraud Dextrose Agar. Inoculation was performed using a sterile bent glass rod, spreading the sample evenly until fully absorbed by the medium. The inoculated plates were incubated at 25 °C for 5 days. After this time, obtained cultures were subjected to macro- and microscopic identification.

## 3. Results

**Figure 1:**
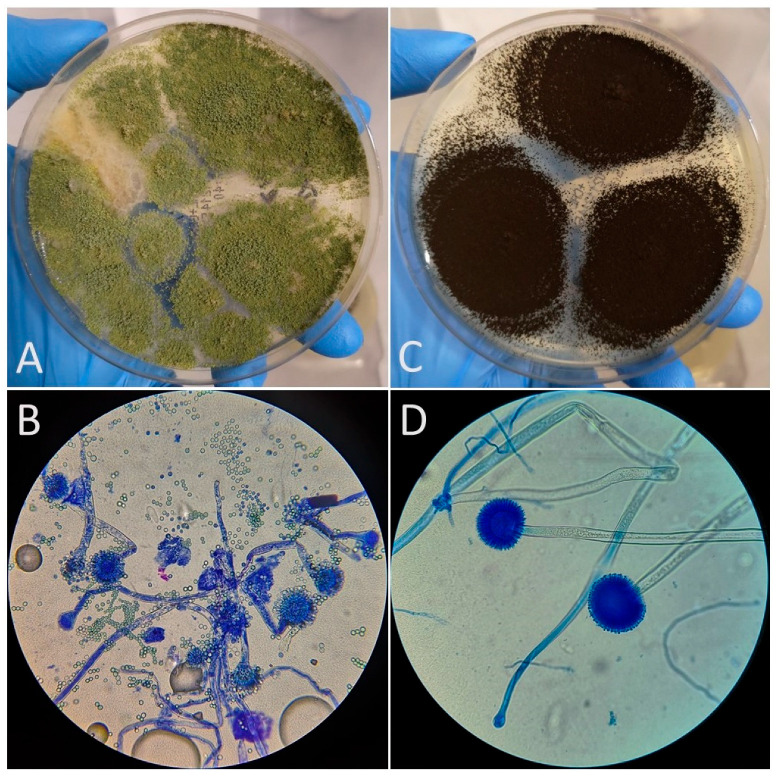
Powdery, olive-green colony on the upper surface of *A. flavus* (**A**) and black-headed conidia colony of *A. niger* (**C**). Microscopic view of *A. flavus*: conidia—radiate heads with loose columns; conidiophores—stipes are hyaline and coarsely roughened (**B**); *A. niger*: conidia—dark, rough-walled, and globose; conidophores—hyaline and smooth-walled (**D**). Conditions: Media: Saburaud DA with chloramphenicol; temperature: 25 °C, humidity: 70%.

**Figure 2:**
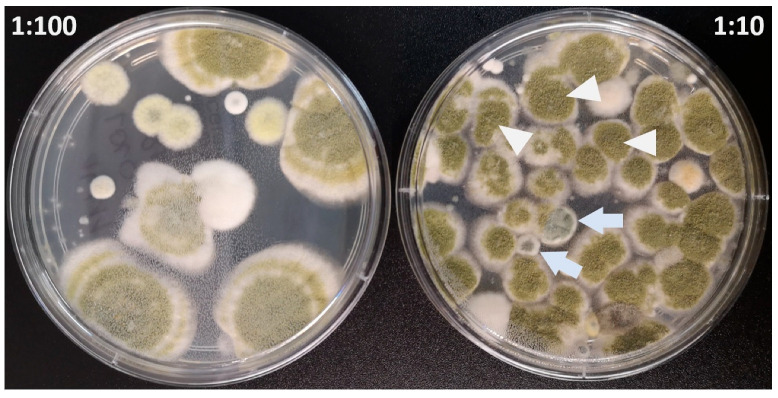
Growth of mold colonies on Sabouraud Dextrose Agar in two dilutions (*A. flavus*—arrowheads, *A. fumigatus*—arrows).

**Figure 3:**
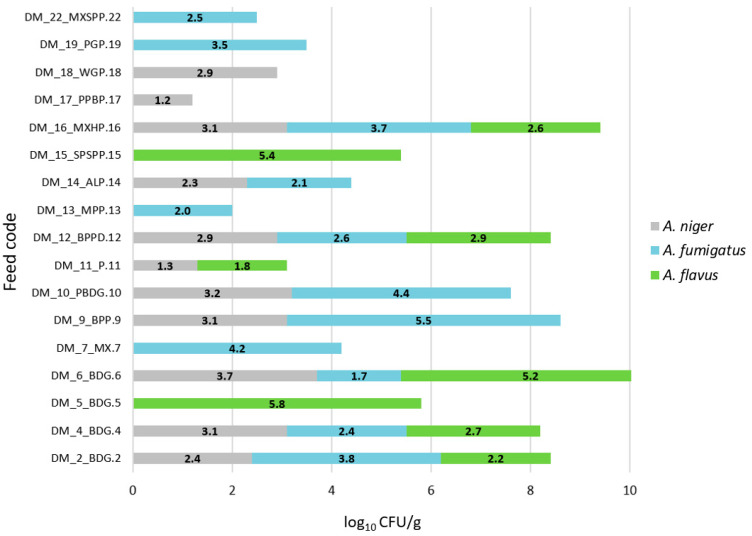
Distribution of *Aspergillus* species identified in analyzed in parrot feed samples.

*Aspergillus* spp. colonies growth was observed in 16 out of 22 feeds (72.7%). The growth of three *Aspergillus* species: *A. niger*, *A. flavus*, and *A. fumigatus* was detected in five (22.7%) feed samples [Figure 1]. Interestingly, in one feed sample, the growth of a *Mucor* species was observed. Figure 2 shows the growth of mold colonies on Sabouraud Dextrose Agar from both dilution (1:10 and 1:100) levels. Table 1 summarizes the proximate composition of the tested feeds, including moisture, protein, fat, fiber, and ash content. The table also includes information on the fungal species isolated from each sample. Figure 3 presents a graphical overview of the quantitative results for *Aspergillus* spp. isolated from the feed samples. Of particular interest are the colony counts exceeding 4 × 10^3^ CFU/g, which were recorded for *A. flavus* in three distinct feed samples, and for *A. fumigatus* in another three samples.

**Figure 4:**
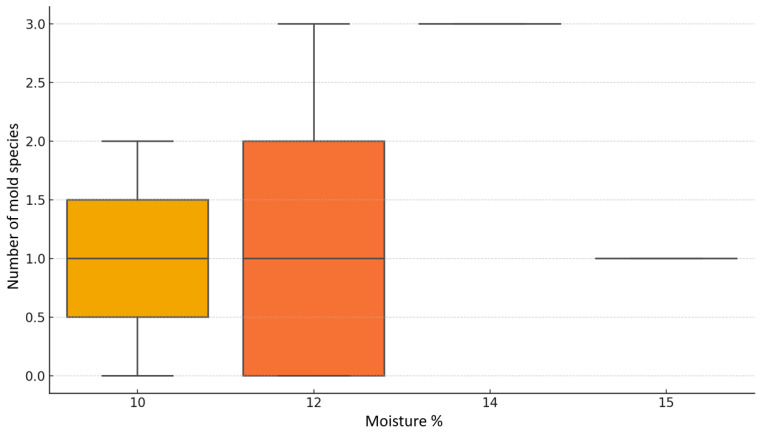
Distribution of mold counts by moisture content.

**Figure 5:**
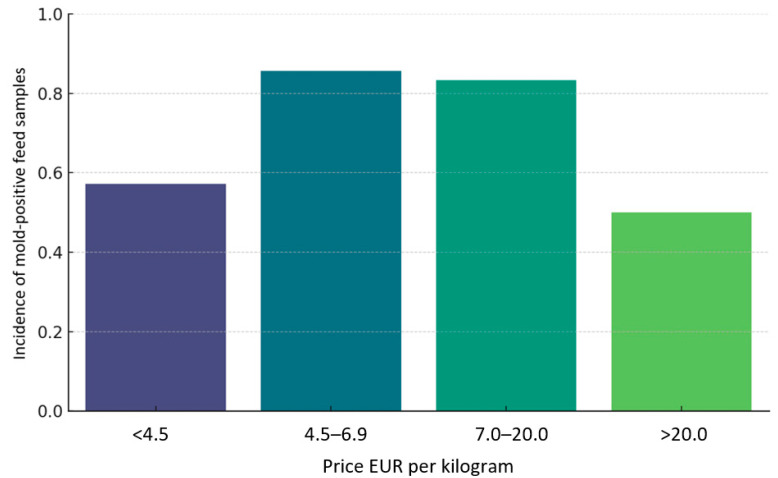
Percentage of parrot feed samples showing mold growth across different price ranges.

A chi-square test of independence was performed to examine the potential relationship between moisture content in parrot feed samples and the number of mold species isolated during microbiological analysis. The test did not reveal any statistically significant association between these two variables, χ^2^(3) = 10.34, *p* = 0.324 [Figure 4]. Although preliminary observations suggested that certain moisture levels might be associated with a higher diversity or frequency of fungal growth, this relationship did not achieve statistical significance within the scope of this study. These findings indicate that, at least in this sample set, moisture content alone cannot be considered a strong or consistent predictor of mold contamination. In terms of economic categorization, the tested feeds were grouped based on their retail price per kilogram into four distinct price brackets. Among these, the mid-range categories, specifically the 4.5–6.9 EUR/kg and 7.0–20.0 EUR/kg groups exhibited the highest proportions of mold contamination, with over 80% of the samples in both groups yielding at least one mold species. This finding raises questions about quality control standards across commonly purchased feed products, particularly those perceived as moderately priced yet widely distributed. By contrast, the lowest frequency of contamination was observed in the highest price category, namely products exceeding 20.0 EUR/kg. However, this group included only a limited number of samples, which restricts the strength of conclusions drawn from this observation. The reduced contamination rate in this premium category may reflect stricter manufacturing standards, enhanced storage protocols, or the inclusion of antifungal treatments, although such hypotheses remain speculative within the context of the current data set. A visual inspection of mold incidence across price categories [Figure 5] supports the impression of variability; however, statistical analysis using a chi-square test again showed no significant association, χ^2^(3) = 2.31, *p* = 0.51.

## 4. Discussion

The microbiological quality of pet food plays a critical role in ensuring animal health. Among the key pathogens detected in dry and wet (canned) pet foods are *Salmonella* spp., *Listeria monocytogenes*, members of the Enterobacteriaceae family, and various mold species [40,41,42,43,44]. The microbiological safety of pet food and feed is most commonly assessed for dogs, cats, livestock, and poultry [43,45,46,47,48].

Fungal contamination of feed with *Aspergillus* spp. and aflatoxins represents a global concern with significant implications for both human and animal health. Commercial dry diets for dogs and cats often incorporate a wide range of agro-industrial ingredients, agricultural by-products, and animal-derived materials [49]. Despite undergoing thermal and mechanical processing during pellet formation, research has demonstrated the presence of fungal genera and species capable of mycotoxin production in these products, posing health risks to companion animals [50,51,52].

To date, research conducted in Poland on fungal contamination and the presence of mycotoxins in commercial pet food has primarily focused on diets formulated for dogs and cats [43,44,53,54]. In contrast, microbiological assessments related to avian species have predominantly concerned poultry feed [25,45,48]. For instance, a study conducted in Iran analyzing 85 poultry feed samples—mainly composed of soy and corn—revealed *Aspergillus* spp. growth in 54 samples, with 20 isolates confirmed as aflatoxin producers [55]. Notably, ingested aflatoxins in poultry can bioaccumulate in eggs, soft tissues, and adipose tissue [56]. Aflatoxin B1 (AFB1) was detected in the livers of quails, ducks, laying hens, and broilers that were fed a diet containing 3 ppm of AFB1. In the described experiment, the contaminated feed was administered for a period of 7 days. Additionally, AFB1 was detected in the muscle tissue of quails on the 8th and 11th days of the experiment; however, it remained undetectable in the muscle tissue of the other tested bird species. In laying hens, the albumen of eggs contained concentrations of free AFB1 metabolites comparable to those found in the liver between the 2nd and 7th days of the experiment [56]. The addition of aflatoxin in the diet of broiler breeder hens has been shown to induce significant immunological impairments in their progeny. This immune dysfunction was manifested through a marked reduction in the phagocytic activity of macrophages against injected sheep red blood cells (SRBC), indicating a compromised innate immune response. Furthermore, a decrease in the production of reactive oxygen intermediates (ROI) by macrophages was observed, suggesting an impaired ability to mount an effective oxidative burst [57]. These findings highlight the transgenerational effects of maternal aflatoxin exposure, emphasizing its potential to adversely affect the immunocompetence of offspring and increase their susceptibility to infectious diseases during early development.

Given that aflatoxins are considered among the most potent hepatocarcinogens and may be transmitted to humans via contaminated eggs, the implementation of stringent monitoring of mold and mycotoxin presence in poultry feed is essential for public health protection [58,59]. The ingestion of high dose of aflatoxins can promote the cytotoxic lysis of red blood cells, which was already confirmed in tests on dogs, rabbits, and poultry [60,61,62]. Anemia is commonly observed in individuals chronically exposed to aflatoxin intoxication, primarily due to impaired iron metabolism. This condition arises from a combination of reduced intestinal iron absorption, suppressed erythropoiesis, and decreased iron availability resulting from the upregulation of hepcidin. These mechanisms are closely associated with chronic inflammation, including aflatoxin-induced enteropathy, which disrupts normal gastrointestinal function and contributes to systemic inflammatory responses [63].

In pregnant animals, exposure to aflatoxins causes decreased live births, low litter size, and fetal anomalies. Wangikar et al. have documented a decreased percentage of live fetuses, increased fetal resorption, impaired organ development, and skeletal anomalies including incomplete scull, ossification, caudal vertebrae agenesis, and bent metacarpals [64]. Exposure to aflatoxins during pregnancy can induce profound morphological and physiological alterations in maternal tissues and organs. In pregnant sows administered aflatoxin B1 at doses of 1 and 3 ppm, histopathological analysis revealed significant hepatic damage, including bile duct epithelial hypertrophy, dissociation of hepatic cords, karyomegaly, and extensive fibrosis. In some regions, this fibrosis led to the formation of pseudolobules and the development of frank adenomas. The spleen exhibited marked lymphocyte depletion within the germinal centers, indicating immunosuppression. Renal lesions were also evident, characterized by intertubular hemorrhages and glomerular atrophy. These findings underscore the hepatotoxic and nephrotoxic potential of aflatoxin B1 during gestation, highlighting its capacity to disrupt organ integrity and function in exposed sows [65].

In wild avifauna, consumption of mold-contaminated grain and grain containing aflatoxins has been identified as a major cause of toxicosis-related mortality [4,5,66]. One limitation of the present study is the lack of assessment of the aflatoxin-producing capacity among the isolated molds. Nevertheless, the detection and identification of *Aspergillus* spp. alone indicates a potential health risk to birds due to their pathogenic and toxigenic nature. Supporting this concern, a study from Nigeria involving 242 samples of feed for caged birds reported the highest incidence of mold contamination with *A. niger* (38.8%) and *A. flavus* (30.1%) [67]. In our investigation, *A. flavus*, *A. niger*, and *A. fumigatus* were identified in 31.8%, 50.0%, and 54.5% of the samples, respectively. Overall, fungal growth was detected in 16 out of 22 samples (72.7%). Only one feed sample (4.5%) showed the presence of *Mucor* spp., with no co-occurrence of other fungal species. This finding is consistent with previous studies reporting the presence of *Mucor* in 10% of pet food samples for dogs and cats [44].

The findings of our study should be interpreted within the broader context of nutritional risks commonly encountered in exotic birds. It is well established that dietary issues in these species stem not only from the potential contamination of feeds with mycotoxins, but also from suboptimal nutrient profiles, including deficiencies in essential vitamins and minerals [68]. This is of particular relevance in the case of aflatoxin exposure, where antioxidant micronutrients, mainly vitamins A, C, E, and selenium play a critical protective role in reducing toxic effects of mycotoxins [69].

Previous in vitro and in vivo studies have demonstrated that dietary supplementation with these vitamins, as well as synthetic antioxidants such as butylated hydroxyanisole (BHA) and butylated hydroxytoluene (BHT), can significantly reduce the cytotoxicity and oxidative damage induced by mycotoxins, with a pronounced effect observed in the presence of aflatoxin B1 [69,70]. Moreover, the nutritional composition of widely available seed mixtures for parrots further exacerbates the risk. Such diets are often characterized by a low content of fat-soluble vitamins: A, D, E, and K, while simultaneously containing excessive fat levels [71,72]. This imbalance may contribute to a compromised immune response, reduced antioxidant defense, and increased susceptibility to mycotoxin-induced pathologies, even in the absence of overt clinical symptoms.

Nevertheless, it is important to emphasize that the absence of visible mold growth in feed samples does not necessarily exclude the presence of mycotoxins. Fungal metabolites may persist in feed even in the absence of active fungal colonies, particularly if contamination occurred during earlier stages of production, processing, or storage. This constitutes a notable limitation of our study. No chromatographic analyses which directly detect or quantify mycotoxins in tested feed samples were performed. Additionally, molecular techniques, including PCR-based assays targeting genes involved in mycotoxin biosynthesis pathways, were not employed. Future research should integrate both analytical chemistry and molecular diagnostics to provide a more comprehensive assessment of mycotoxin exposure in avian diets [5,31,73].

## 5. Conclusions

Although preliminary, the findings of this pilot study clearly indicate a potential microbiological hazard associated with commercial parrot feed, particularly due to the frequent presence of *Aspergillus* spp., a genus known to cause life-threatening aspergillosis in psittacine birds. The detection of *A. fumigatus*, *A. flavus*, and *A. niger* in over 70% of tested feed samples raises serious concerns about the microbial safety of diets intended for exotic companion birds. Despite the well-documented susceptibility of psittacine birds to life-threatening aspergillosis and mycotoxicosis, there are currently no established regulatory standards governing permissible levels of fungal contamination in avian feed. Furthermore, specific guidelines addressing acceptable concentrations of molds in commercial diets intended for parrots remain absent, representing a critical gap in feed safety oversight for exotic companion birds. This regulatory gap underscores the urgent need for standardized monitoring and quality control procedures in the production of pet bird diets, particularly those composed of cereal-based ingredients, which are especially prone to fungal colonization. The results presented here highlight the need to raise awareness among veterinarians, breeders, and bird owners regarding the health risks posed by mold-contaminated feed. Given the increasing popularity of parrots driven by their sociability, vocal mimicry, and longevity, the responsibility for ensuring their health and welfare is growing accordingly. We hope that this study provides a broader perspective on the underrecognized risk of aspergillosis in exotic birds and serves as a starting point for further, more comprehensive research. Future efforts should focus on developing microbiological standards for parrot feed and promoting the use of high-quality, preferably human-grade ingredients to minimize the risk of aspergillosis and mycotoxicosis in exotic pet birds.

## Figures and Tables

**Table 1:** Proximate composition of the analyzed parrot feeds and identification of the isolated fungal species.

Feed Code	Price Per Kilogram [EUR]	Moisture [%]	Crude Protein [%]	Fat [%]	Fiber [%]	Ash [%]	*A. niger*	*A. fumigatus*	*A. flavus*	Other Molds	Number of Cultured Molds
DM_1_BDG.1	3.0	12.0	15.0	3.5	12.0	7.0	-	-	-	*Mucor* sp.	1
DM_2_BDG.2	3.4	12.0	11.2	6.0	8.9	5.0	+	+	+	-	3
DM_3_BDG.3	3.4	10.0	12.3	6.3	6.7	3.0	-	-	-	-	0
DM_4_BDG.4	3.8	12.0	9.7	5.8	11.3	5.6	+	+	+	-	3
DM_5_BDG.5	4.2	12.0	12.5	8.0	11.2	6.5	-	-	-	-	0
DM_6_BDG.6	4.2	12.0	11.2	6.0	8.9	4.5	+	+	+	-	3
DM_7_MX.7	4.7	12.0	15.0	13.2	8.0	6.0	-	+	-	-	1
DM_8_NT.8	4.9	12.0	24.2	49.0	8.5	3.0	-	-	-	-	0
DM_9_BPP.9	4.9	10.0	13.0	16.0	15.0	3.0	+	+	-	-	2
DM_10_PBDG.10	4.9	12.0	12.0	5.0	7.5	3.5	+	+	-	-	2
DM_11_P.11	5.1	12.0	12.5	7.0	8.0	6.0	+	-	+	-	2
DM_12_BPPD.12	5.1	12.0	13.0	15.0	10.0	5.0	+	+	+	-	3
DM_13_MPP.13	5.6	12.0	12.0	7.5	11.2	6.2	-	+	-	-	1
DM_14_ALP.14	6.5	12.0	13.8	13.0	13.6	2.5	+	+	-	-	2
DM_15_SPSPP.15	7.0	12.0	12.6	7.0	8.5	4.9	-	-	+	-	1
DM_16_MXHP.16	7.7	14.0	16.6	31.0	16.0	3.6	+	+	+	-	3
DM_17_PPBP.17	7.7	12.0	13.2	14.7	14.0	3.5	+	-	-	-	1
DM_18_WGP.18	7.7	15.0	11.0	4.0	10.0	5.8	+	-	-	-	1
DM_19_PGP.19	9.8	15.0	12.5	4.5	9.7	3.0	-	+	-	-	1
DM_20_PRMB.20	10.5	12.0	13.0	18.0	20.0	3.3	-	-	-	-	0
DM_21_BPPT.21	23.4	12.0	12.0	6.0	2.0	3.1	-	-	-	-	0
DM_22_MXSPP.22	105.0	12.0	11.0	19.0	13.0	3.0	-	+	-	-	1

## Data Availability

The original contributions presented in this study are included in the article. Further inquiries can be directed to the corresponding author.
